# Re-thinking cluster policies: the role of shared vision and Place Leadership on the development of resilient clusters

**DOI:** 10.1365/s42681-023-00032-9

**Published:** 2023-05-10

**Authors:** Patricia Ganske, Claus-Christian Carbon

**Affiliations:** University of Bamberg, Bamberg, Bavaria Germany

**Keywords:** Cluster policy, Cluster development, Place Leadership, Shared vision, Regional development, Policy making, Openness, Cooperation

## Abstract

Facing increasing interregional and international competition, the need for innovating economic and socio-institutional structures is virulent. One possibility is establishing clusters to promote the idea that developing a network of companies with a mix of cooperative and competitive practices leads to competitive advantages for the respective area. Organizing clusters means developing a cluster culture, a communication strategy and a selection criterion for creating the desired mix of companies. We base our opinion paper on an extensive literature review of scientific sources about clusters, cluster policy and Place Leadership. We reveal that the prevailing cluster policy often does not lead to sustainably successful clusters. We conclude that we need a shared vision among cluster members to develop resilient clusters. Shared visions create a sense of belonging, social identity, and internalization of values and attitudes. This way, it is possible that a sustainable and trust-oriented network structure can develop, and knowledge spillovers within the cluster, necessary for resilient clusters, can occur. True Place Leadership is crucial to deal with the needed diversity of actors and stakeholders, some of whom are in competition, inspiring, motivating, and guiding shared, cooperative, and collaborative ways of working. Employing Place Leadership can outperform current policy measures in other regional, industrial and structural areas and might even make them redundant.

## Introduction

Hardly any other policy concept has made such an impressive triumph in political practice in a relatively short time as the cluster concept propagated by Porter (1998). Porter’s cluster theory has become the standard concept in the field as a tool for promoting national, regional and local competitiveness, innovation, as well as urban and regional growth. This theory acts as a generator that provides potential advantages in perceiving both the need and the opportunity for innovation (Porter and Stern 2001). Clusters have become an object of desire for many cities and regions based on the widely accepted assumption that increased specialization leads to higher levels of productivity, growth, and employment (Steiner 1998). Experts state that clusters positively impact the economy’s development through affecting productivity, innovation and entrepreneurship (Kozonogova et al. 2019). The cluster concept is based on the idea that an agglomeration of firms developing a network of relationships and a subtle mix of cooperative and competitive practices leads to the creation of competitive advantages for the area in which this agglomeration is located. The cluster approach is applied at various geographic levels, such as within city districts, and at the regional, national or even international level (Cumbers and McKinnon 2004). Internationally known flagship clusters such as Silicon Valley that have shown sustainable success have attracted policy interest and called for the promotion of clusters worldwide. Porter’s concept, which was initially based more on microeconomic competition analysis and not aimed at state intervention in the market, gradually changed policy practice into a “directive“ for structural and innovative policy action (Rothgang and Lageman 2011).

Cluster policies try to promote the supply of local and regional public goods which are absent due to market failure (Martin and Sunley 2003). However, in practice, it is often the case that new clusters emerge or even existing ones change their structure, among other things, in connection with the conditions of the cluster programs announced by the public funding agencies. The cluster organizations align themselves with the respective goals of the funding agencies. This applies to the content and strategic orientation, but also in part to the membership structure and regional expansion, which are aimed at the respective program (Rothgang and Lageman 2011). As a result, clusters that are not sustainably successful because they lack the necessary framework conditions are implemented and promoted. These basic conditions include scientific and technological excellence of the actors involved, openness to new cluster members and technologies, a grown and trust-oriented network structure, and the mapping of complete value chains and chains of knowledge production (Bührer et al. 2003).

Visions and shared visions are generally developed to create motivation to move from a current state to the desired end state, whether on the individual, team or organizational level (Boyatzis et al. 2015). A shared vision between cluster members can influence the enabling conditions for resilient clusters. However, the leadership of place-based partnerships is distinct from the leadership of organizations (Bowden and Liddle 2018). In the regional context, one is in a multi-agent, multi-objective, multi-vision and pluralistic process. Therefore, a leader who creates a shared vision for the region and the cluster is needed — a Place Leader.

With this opinion essay, we aim to provide insights into what we see as current ineffective cluster policies. Using evidence from the literature on clusters, cluster policy, and Place Leadership, we argue in the following that only true Place Leadership is capable of dealing with a variety of actors and stakeholders, some of whom compete. Based on a shared vision, affiliated actors will inspire, motivate and collaborate and can thereby achieve a sustainable and trust-oriented network structure, creating knowledge spillovers within the cluster, which is necessary for resilient clusters. Employing Place Leadership can outperform and possibly even supersede current policies in other regional, industrial, and structural areas.

## Cluster policies on the wayside

Cluster policy is not a policy field on its own but rather at the interface of established policy fields such as regional, industrial, and structural policy, regional and municipal economic stimulation, technology and innovation policy, as well as science and research policy. Therefore, clearly defining cluster policy can be an issue, as it is difficult to distinguish it from other policies in a given economy (Jankowiak 2019). Cluster policies can vary in scope and design, but they generally refer to a particular model and conceptual repertoire which is rooted in innovation studies. Consequently, they rely on a ”functional model of governance” established in the academic literature (Blümel 2021). Cluster policy combines its instruments in an innovative and cluster-focused way (Ahn 2018) and has emerged as an archetype for a group of “soft” policies that aim to achieve relational outcomes (Burfitt and MacNeill 2008). However, cluster policies are not easy to develop and implement because they require a fundamental understanding of the specific problems within each cluster (Grashof 2020). Four fields of cluster policy can be identified: Support for network initiation and -management, joint marketing of an industrial specialism based on location marketing and raising awareness of the region’s industrial strengths, services for cluster members such as funding advice, marketing or design services and the identification and elimination of weaknesses in existing cluster value chains. According to the Commission of the European Communities (17 Oct 2008) cluster policy should aim to increase socio-economic benefits by promoting existing clusters or creating favorable conditions for forming new clusters.

In recent years, complaints about the actual economic relevance and efficiency of regional innovation policies, such as cluster policy, have increased (Grashof 2020; Lehmann and Menter 2018). Current cluster policies are often characterized by highly standardized measures that may work in some cases but not in all (Zenker et al. 2019). Contrary to the objective of the Commission of the European Communities (17 Oct 2008), in practice, sector- and technology-specific funding was often tendered at the state and federal levels (e.g. cluster *automotive-bw* (2010), funding of new clusters within the *Guideline to promote the use and construction of demonstration plants and example regions for the industrial bioeconomy* (2021), funding for regional innovation networks *Future Cluster Initiative* (2019)). Politics thus expanded its competencies from a supporting role to creating the best possible framework conditions for the emergence of clusters and actively interfered in market activities. The pressure to conform in order to obtain funding leads cluster organizations to adapt to the respective program documents in terms of technological orientation, membership structure, strategy, and regional concentration. The more concretely formulated these are, the stronger the focus required to acquire the funding (Rothgang and Lageman 2011). This approach becomes particularly problematic when trying to implement clusters not based on region-specific cluster potentials, a phenomenon increasingly occurring in European countries in which many artificial cluster initiatives are created as a result of entrepreneurial efforts to obtain public funding, but which do not represent true clusters in the sense of actual market structures with strong regional specialization (Kowalski 2020). Such problematic linkages are also promoted by the fact that, according to Martin and Sunley (2003), policymakers are under pressure to find clusters in as many regions as possible to avoid offending regional interests—often such linkages lead to inefficient communication and collaboration. There is a consensus that cluster promotion policies are unlikely to succeed in creating clusters ab initio (Schmitz and Nadvi 1999).

As Grashof (2020) also points out, we believe that cluster policy should instead focus on the specific conditions and needs within regional clusters. Cluster policies should attempt to build on the potential already present in a particular economy and not provide “watering-can-like” false incentives to implement new clusters that correspond to the funding calls. The current approach to cluster policy, which is often not based on regional specifics and is standardized, runs the risk of leading to idealized or preconceived outcomes that all institutions can subscribe to, but which have little to do with actual local economic processes or potential (Burfitt and MacNeill 2008; Zenker et al. 2019). Since policymakers cannot support all clusters and technologies in the same way, it has to decide which ones to pay special attention to and which ones do not. Policymakers usually rely on economic and scientific arguments when deciding in favor of certain clusters. However, these arguments can be criticized; they are often not scientifically based but rather political while ignoring economic warnings and historical evidence against selective support (Hospers et al. 2008). Policy responses and associated decisions to rapidly changing political, economic, social, technological, and environmental conditions are often reactive rather than proactive and fail or prove inadequate (Sotarauta et al. 2017). There are no fundamental reasons to believe that policymakers are better able to predict the future economic potential of specific ventures (including clusters) than entrepreneurs. Given the inherently uncertain nature of new technologies, such government failure is likely, especially when it comes to high-tech clusters.

It has become increasingly apparent that the question of sustainability or continuity of development must be raised in the discussion about cluster and network promotions as practiced today. Whether cluster or network initiatives survive in the long term depends on their economic sense and the financial strength of the companies involved. It is commonly assumed that after an initial phase supported by state funding, cluster organizations will continue to develop on their own without state funding. Experience shows that continuous development is only likely in specific fields where financially strong large companies are present and strongly committed (Rothgang and Lageman 2011). Companies are only willing to invest in a cluster if they get a corresponding return and the investment is seen as necessary to achieve a particular goal. If the goal of the cluster, which the policymakers appoint, does not correspond to the goal of the cluster members, they will probably not invest. Thus it happens repeatedly that the activities of a cluster are discontinued after public funding has expired without achieving a lasting result (Graf and Broekel 2020). This must be critically questioned in terms of the benefit-cost ratio and a possible waste of resources.

It turns out that policies to promote clusters often amount to “picking winners or backing losers” (Norton 1999, non-paginated). This is because governments are not in a position to generate the knowledge that would be required to make most clusters work. As public choice theory makes clear, “government failure” is just as common as “market failure”, due to massive information asymmetries and strategic behavior by politicians and bureaucrats (Wolf 1990). There is no reason to believe that institutional arrangements where governments actively promote clusters will generate relevant knowledge. Thus, even if governments were made up of people with only the common good in mind, they could not implement successful cluster policies.

## Shared vision as a cluster promoter

Considering a cluster, we find ourselves in a regional context characterized by multi-agent, multi-objective, multi-vision, and pluralistic processes. The management of clusters is therefore associated with particular difficulties for those responsible. With the large number of actors involved in a cluster, it is often a significant challenge to balance the different cultures and institutional agendas and to find common ground and mutual benefit (Burfitt and MacNeill 2008). Critical factors for the sustainable success of a cluster include framework conditions such as a grown and trust-oriented network structure and openness to new cluster members and technologies (Bührer et al. 2003). Cluster companies must set rival thinking aside in favor of a shared vision. These requirements cannot be created or enforced by cluster policy but must emerge from within the cluster through the willingness and commitment of the cluster members.

Cluster innovation, and thus resilient clusters, can only be achieved if members are willing to collaborate by conviction, which requires that individuals identify with the group (in this case, the cluster) and thus base their self-concept and self-esteem in part on belonging to the group (cluster) (Moriano et al. 2014). Hence, cluster performance is influenced by cluster identification, which in turn is shaped by cluster leadership (Chen et al. 2018).

Visions motivate people to act and inspire them to go beyond their present state. Deeper than goals or strategies, desired images of the future, or the hoped-for future, can provide a sense of mission (Boyatzis et al. 2015). A shared vision contributes to cluster members identifying with clusters. Griffin et al. (2010) found that leaders who developed a vision achieved more openness and adaptability in organizations. Likewise, this can be applied to companies in a cluster context. A shared vision within a cluster thus represents the fundamental prerequisite for clusters to be long-term successful. On the one hand, a shared vision ensures in the short and medium run that competitive thinking is put aside in favor of cluster thinking and that clusters are implemented which build on a certain potential already existing in the economy. On the other hand, it ensures that in the long run, the continuation of a cluster is also guaranteed without financial support through subsidies from cluster policy. This also ensures that clusters can continue to develop independently and proactively adapt to the market without being exposed to the danger of possible misalignment or log-in through political requirements.

## Place Leader—the initiator of a shared vision

Place Leadership is defined as “the mobilization and coordination of diverse groups of actors to achieve a collective effort aimed at enhancing the development of a specific place” (Sotarauta 2021, p.152). In contrast to the association of a leader as “the one who makes it happen”, where leadership is viewed as an individual ability to direct others on what to do based on strong hierarchical relationships in decision making and formal power, Place Leadership is often referred to as shared, cooperative, or collaborative due to the challenge of dealing with a variety of actors and stakeholders (Horlings et al. 2018). Place Leaders are usually not assigned leaders by position but leaders by personality. Place Leaders do not hold a formal position that allows them to execute power over others. They gain power and leadership positions by influencing and convincing others (Sotarauta 2016; Beer et al. 2019). It is important to note that in addition to individuals from various sectors, including government, business, academia, and the community, institutions and organizations can also take on the role of Place Leaders. Many institutions and organizations play such a leading role in shaping the development and growth of a particular place. For example, a chamber of commerce, local economic development agency, or non-profit organization can take a leadership role by advocating for policies and investments that support the development of the place they serve, fostering collaboration and partnerships among stakeholders, and supporting a shared vision for the future of the place. Place Leaders are widely believed to have an important impact on the economic and social performance of regions, for example, by helping unlocking a region from its path and guiding it in new directions (Sotarauta and Beer 2017).

Place Leaders gain their influence by stimulating imagination, (re)framing issues, and developing new agendas, helping to “think the unthinkable“ (Horlings et al. 2010). Rather than trying to convince all stakeholders of the one “right and true“ view, Place Leaders strive to create a shared vision for the place among all stakeholders. They work as a kind of connector between the different stakeholders (Horlings et al. 2018) and try to strengthen the sense of place and identification utilizing a shared vision and thus develop the place. Place Leaders moderate the process of creating a shared vision among all cluster members. In this way, they indirectly influence the framework for implementing and managing resilient clusters.

## Conclusion

The experience of the past years has shown that cluster policy, as it has been pursued in many cases in recent years, often does not lead to sustainably successful clusters. The “watering-can-like” approach of industry- and technology-specific cluster support programs without a specific focus on the cluster potential available in the regions led to misallocations of clusters, with the result that these were discontinued when the state subsidies expired. To make cluster policy tailored and thus sustainable, it would be necessary to identify a particular cluster or cluster potentials and its components within a local economy and develop a picture of the nature of the interactions among its actors, as well as an understanding of the nature of the market failures it faces (Burfitt and MacNeill 2008). However, this poses significant and almost insurmountable challenges for policymakers due to a lack of information and metrics.

Governments pursuing cluster policies should shift their attention from targeting and subsidizing specific industries and technologies to facilitating the development and functioning of clusters in the economy, thus also considering the Commission of the European Communities’ (17 Oct 2008) demands on cluster policies. In the literature, this desired change in policy orientation is described as a shift from specific to general policy, from direct to indirect intervention, and from vertical to horizontal policy (Chang 1994; McDonald and Dearden 2005). In essence, it should be left to the market itself to determine which clusters are formed regionally. After all, clustering is ultimately the result of entrepreneurial activity and is driven by the production of goods and services to generate profits. The state cannot sufficiently replace the market in the formation of these clusters (Sautet 2002).

Resilient clusters are not created by cluster policies that intervene in the market. What is needed for a cluster to be sustainably successful, which also means adapting to the changing demands of the market, is a shared vision among the cluster members. Only through sharing a vision, the consequent recognition of the benefits of the cluster initiative will be that companies often competing outside the cluster project are willing to open up to the cluster members. A shared vision is the only way to create a trust-based network structure—and only on trust can we build up sustainable cluster activities. Therefore, the necessary knowledge spillovers within the cluster can occur and the cluster can thrive. Especially in times when regional economies are faced with the task of transforming their economic structures due to recurring economic crises, globalization, digitalization, climate change, the depletion of natural resources and a variety of other societal dynamics such as migration and demographic change and, more recently, the COVID-19 pandemic or belicistic conflicts among neighbor countries, clusters can make a significant contribution to ensuring that a region successfully overcomes these challenges. A shared vision, the associated open-mindedness toward new technologies and the opportunity for intensive exchange and resulting innovations not only lead the way to the resilience of clusters, but also to the sustainable economic success of regions.

The process of establishing and implementing a common vision is facilitated by Place Leaders, who are the link between the various interests of the cluster members. Since Place Leaders have no formal power on the cluster members and can neither pass guidelines nor create monetary incentives, clusters operated by Place Leaders will have the critical qualitative mass to initiate and promote sustainable networks as they are not merely attracted by false or misleading inducements.

In conclusion, future cluster policy should return to the horizontal measures of cluster promotion and not take an active role in the initiation of clusters. Future scientific work should consider the importance of a shared vision among cluster members when analyzing cluster formation, development and governance. It should also be questioned to what extent effective Place Leadership and a shared vision could outperform current policy measures in other regional, industrial and structural areas and might even make them redundant.

## Electronic supplementary material

Below is the link to the electronic supplementary material.

Supplementary Material 1
