# Supplementary material for: Re-thinking cluster policies: the role of shared vision and Place Leadership on the development of resilient clusters
Source: Leadersh Educ Personal Interdiscip J. 2023 May 10;4(1-2):1–6. [Article in German] doi: 10.1365/s42681-023-00032-9 (PMC10171138; doi:10.1365/s42681-023-00032-9)
Supplement: Supplementary file 1 — Supplementary Material 1 [file 42681_2023_32_MOESM1_ESM.pdf]

Formatvorlagendefinition: Kommentarzeichen

Re-thinking cluster policies:

The role of shared vision and ~~place leadership~~Place Leadership on the development of resilient clusters

Number of words: ~~2,781~~3,200

Short abstract (~~185~~213 words)

~~Against~~Facing increasing interregional and international competition, the ~~pressure increases to transform~~need for innovating economic ~~structures~~ and ~~renew the~~ socio-institutional ~~fabric. Policymakers have addressed these requirements by promoting the establishment and development of~~structures is virulent. One possibility is establishing clusters~~. The cluster concept is based on~~ to promote the idea that ~~an agglomeration of firms~~ developing a network of ~~relationships and~~companies with a ~~subtle~~ mix of cooperative and competitive practices leads to competitive advantages for the respective area. ~~Cluster policy tries to support the creation of~~ Organizing clusters~~, influences the content~~ means developing a cluster culture, a communication strategy and ~~strategic orientation of the clusters, as well as the~~ a selection ~~of members and regional scope. This~~ criterion for creating the desired mix of companies. We base our perspective article ~~suggests~~on an extensive literature review of scientific sources about clusters, cluster policy and Place Leadership. We revealed that the prevailing cluster policy often does not lead to sustainably successful clusters. We conclude that we need a shared vision among cluster members to develop resilient clusters. ~~We argue that such a vision~~Shared visions create a sense of belonging, social identity, and internalization of values and attitudes. This way, it is possible that a sustainable and trust-oriented network structure can ~~only be achieved by true Place Leadership capable of dealing~~develop, and knowledge spillovers within the cluster, necessary for resilient clusters, can occur. True Place Leadership is crucial to deal with ~~a variety~~the needed diversity of actors and stakeholders, some of whom are in competition, inspiring, motivating, and guiding shared, cooperative, and collaborative ways of working. Employing Place Leadership can outperform current policy measures in other regional, industrial and structural areas and might even make them redundant.

Formatiert: Englisch (Vereinigte Staaten)

Keywords: Cluster policy, cluster development, shared vision, ~~place leadership, leadership~~Place Leadership, regional development, policy making, openness
, cooperation

Formatiert: Fußzeile, Zeilenabstand: Doppelt

Introduction

Hardly any other policy concept has made such an impressive triumph in political practice in a relatively short time as the cluster concept propagated by Porter (1998). Porter's cluster theory has become the standard concept in the field as a tool for promoting national, regional and local competitiveness, innovation, as well as urban and regional growth by acting. This theory acts as a generator that provides potential advantages in perceiving both the need and the opportunity for innovation (Porter and Stern, 2001). Clusters have become an object of desire for many cities and regions based on the widely accepted assumption that increased specialization leads to higher levels of productivity, growth, and employment (Steiner, 1998). Experts state that clusters positively impact the economy's development through affecting productivity, innovation and entrepreneurship (Kozonogova et al., 2019). The cluster concept is based on the idea that an agglomeration of firms developing a network of relationships and a subtle mix of cooperative and competitive practices leads to the creation of competitive advantages for the area in which this agglomeration is located. The cluster approach is applied at various geographic levels, such as within city districts, and at the regional, national or even international level (Cumbers and McKinnon, 2004). The promotion of Internationally known flagship clusters such as Silicon Valley hasthat have shown sustainable success have attracted policy interest because of and called for the success stories promotion of clusters such as Silicon Valley. Porter'sworldwide. Porter's concept, which was initially based more on microeconomic competition analysis and not aimed at state intervention in the market, gradually changed in policy practice into a "directive" for structural and innovationinnovative policy action (Rothgang and Lageman, 2011).

Cluster policies try to promote the supply of local and regional public goods which are, absent due to market failure , absent (Martin and Sunley, 2003).. However, in practice, it is often the case that new clusters emerge or even existing ones change their structure, among other things, in connection with the conditions of the cluster programs announced by the public funding agencies. The cluster organizations align themselves with the respective goals of the funding agencies. This applies to the content and

strategic orientation, but also in part to the membership structure and regional expansion, which are aimed at the respective program (Rothgang and Lageman, 2011). As a result, clusters that are not sustainably successful because they lack the necessary framework conditions (including theare implemented and promoted. These basic conditions include scientific and technological excellence of the actors involved, openness to new cluster members and technologies, a grown and trust-oriented network structure, and the mapping of complete value chains and chains of knowledge production (see Bührer et al., 2003)) are implemented and promoted.).

Visions and shared visions are generally developed to create motivation to move from a current state to the desired end state, whether it be on the individual, team or organizational level (Boyatzis et al., 2015). A shared vision between cluster members can influence the enabling conditions for resilient clusters. However, the leadership of place-based partnerships is distinct from the leadership of organizations (Bowden and Liddle, 2018). In the regional context, one is in a multi-agent, multi-objective, multi-vision and pluralistic process. Therefore, a leader is needed who creates a commonshared vision for the region and the cluster located there is needed — a place leaderPlace Leader.

With this opinion essay, we aim to provide insights into what we see as current ineffective cluster policies. Using evidence from the literature on clusters, cluster policy, and Place Leadership, we argue in the following that only true Place Leadership is capable of dealing with a variety of actors and stakeholders, some of whom compete. Based on a shared vision, affiliated actors will inspire, motivate and collaborate and can thereby achieve a sustainable and trust-oriented network structure, creating knowledge spillovers within the cluster, which is necessary for resilient clusters. Employing Place Leadership can outperform and possibly even supersede current policies in other regional, industrial, and structural areas.

Cluster Policies on the Wayside

Cluster policy is not a policy field on its own but rather at the interface of established policy fields such as regional, industrial, and structural policy, regional and municipal economic stimulation, technology and innovation policy, as well as science and research policy. Therefore, clearly defining cluster policy can be an issue, as it is difficult to distinguish it from other policies in a given economy (Jankowiak, 2019). Cluster policies can vary in scope and design, but they generally refer to a particular model and conceptual repertoire which is rooted in innovation studies. Consequently, they rely on a ”functional model of governance” established in the academic literature (Blümel, 2021). Cluster policy combines its instruments in an innovative and cluster-focused way (Ahn, 2018) and has emerged as an archetype for a group of “soft” policies that aim to achieve relational outcomes (Burfitt and MacNeill, 2008). However, cluster policies are not easy to develop and implement because they require a fundamental understanding of the specific problems within each cluster (Grashof, 2020). Four fields of cluster policy can be identified: Support for network initiation and -management, joint marketing of an industrial specialism based on the basis of location marketing and raising awareness of the region'sregion’s industrial strengths, services for cluster members such as funding advice, marketing or design services and the identification and elimination of weaknesses in existing cluster value chains. According to the Commission of the European Communities (17.10. Oct 2008) cluster policy should aim to increase socio-economic benefits by promoting existing clusters or creating favorable conditions for the formation offorming new clusters.

Contrary to this objectiveIn recent years, complaints about the actual economic relevance and efficiency of regional innovation policies, such as cluster policy, have increased (Grashof, 2020; Lehman and Menter, 2018). Current cluster policies are often characterized by highly standardized measures that may work in some cases but not in all (Zenker et al., 2019). Contrary to the objective of the Commission of the European Communities (17 Oct 2008), in practice, sector- and technology-specific funding was often tendered at the state and federal levels (e.g. cluster *automotive-bw* (2010), funding of new clusters within the *Guideline to promote the use and construction of demonstration plants and example regions for the industrial bioeconomy* (2021), funding for regional innovation networks *Future Cluster Initiative* (2019)).

Politics thus expanded its competencies from a supporting role to creating the best possible framework conditions for the emergence of clusters and actively interfered in market activities. The pressure to conform in order to obtain funding leads cluster organizations to adapt to the respective program documents in terms of technological orientation, membership structure, strategy, and regional concentration. The more concretely these are formulated these are, the stronger the focus required to successfully acquire the funding (Rothgang and Lageman, 2011). This approach becomes particularly problematic when trying to implement clusters not based on region-specific cluster potentials., a phenomenon increasingly occurring in European countries in which many artificial cluster initiatives are created as a result of entrepreneurial efforts to obtain public funding, but which do not represent true clusters in the sense of actual market structures with strong regional specialization (Kowalski, 2020). Such problematic linkages are also promoted by the fact that, according to (Martin and Sunley, 2003), policymakers are under pressure to find clusters in as many regions as possible to avoid offending regional interests—often such linkages lead to inefficient communication and collaboration. There is a consensus that cluster promotion policies are unlikely to succeed in creating clusters ab initio (Schmitz and Nadvi, 1999).

As Grashof (2020) also points out, we believe that cluster policy should instead focus on the specific conditions and needs within regional clusters. Cluster policies should attempt to build on the potential already present in a particular economy and not provide "watering-can-like" false incentives to implement new clusters that correspond to the funding calls. The current open approach to cluster policy, which is often not based on regional specifics and is standardized, runs the risk of leading to idealized or preconceived outcomes that all institutions can subscribe to, but which have little to do with actual local economic processes or potential (Burfitt and MacNeill, 2008).; Zenker et al., 2019). Since policymakers cannot support all clusters and technologies in the same way, it has to decide which ones to pay special attention to and which ones do not. When deciding in favor of certain clusters, policymakersPolicymakers usually rely on economic and scientific arguments. when deciding in favor of certain clusters. However,

these arguments can be criticized; often they are often not scientific at allscientifically based but rather political while ignoring economic warnings and historical evidence against selective support (Hospers et al., 2008). Frequently, policyPolicy responses and associated decisions to rapidly changing political, economic, social, technological, and environmental conditions are often reactive rather than proactive and fail or prove inadequate (Sotarauta et al. 2017). There are no fundamental reasons to believe that policymakers are better able to predict the future economic potential of specific ventures (including clusters) than entrepreneurs. Given the inherently uncertain nature of new technologies, such government failure is likely, especially when it comes to high-tech clusters.

It has become increasingly apparent that in the discussion about cluster and network promotions as practiced today, the question of sustainability or continuity of development must be raised. in the discussion about cluster and network promotions as practiced today. Whether cluster or network initiatives survive in the long term depends not only on their economic sense but also onand the financial strength of the companies involved. It is commonly assumed that after an initial phase supported by state funding, cluster organizations will continue to develop on their own without state funding. Experience shows that continuous development is only likely in specific fields where financially strong large companies are present and strongly committed (Rothgang and Lageman, 2011). Companies are only willing to invest in a cluster if they get a corresponding return and the investment is seen as necessary to achieve a particular goal. If the goal of the cluster, which the policymakers appoint, does not correspond to the goal of the cluster members, they will probably not invest. Thus it happens repeatedly that the activities of a cluster are discontinued after public funding has expired without achieving a lasting result. (e.g. Graf and Broekel, 2019). This must be critically questioned in terms of the benefit-cost ratio and a possible waste of resources.

It turns out that policies to promote clusters often amount to “picking winners or backing losers” (Norton, 1999, non-paginated). This is because governments are not in a position to generate the knowledge that would be required to make most clusters work. As public choice theory makes clear, “government

failure" is just as common as "market failure", due to massive information asymmetries and strategic behavior by politicians and bureaucrats (Wolf, 1990). There is no reason to believe that institutional arrangements where governments take an active role in promotingactively promote clusters will generate relevant knowledge. Thus, even if governments were made up of people with only the common good in mind, they wouldcould not be able to implement successful cluster policies.

Shared Vision as a cluster promoter

Considering a cluster, we find ourselves in a regional context characterized by multi-agent, multi-objective, multi-vision, and pluralistic processes. The management of clusters is therefore associated with particular difficulties for those responsible. With the large number of actors involved in a cluster, it is often a significant challenge to balance the different cultures and institutional agendas and to find common ground and mutual benefit (Burfitt and MacNeill, 2008). Critical factors for the sustainable success of a cluster include framework conditions such as a grown and trust-oriented network structure and openness to new cluster members and technologies (see Bührer et al., 2003). Cluster companies must set rival thinking aside in favor of a shared vision. These requirements cannot be created or enforced by cluster policy but must emerge from within the cluster through the willingness and commitment of the cluster members.

Cluster innovation, and thus resilient clusters, can only be achieved if members are willing to collaborate by conviction, which requires that individuals identify with the group (in this case, the cluster) and thus base their self-concept and self-esteem in part on belonging to the group (cluster) (Moriano et al., 2014). Hence, cluster performance is influenced by cluster identification, which in turn is shaped by cluster leadership (Chen et al., 2018).

Visions motivate people to act and inspire them to go beyond their present state. Deeper than goals or strategies, desired images of the future, or the hoped-for future, can provide a sense of mission (Boyatzis

et al., 2015). A shared vision contributes to cluster members identifying with clusters. (Griffin et al., 2010) found that leaders who developed a vision achieved more openness and adaptability in organizations. Likewise, this can be applied to companies in a cluster context. A shared vision within a cluster thus represents the ~~basic~~fundamental prerequisite for clusters to be long-term successful. On the one hand, a shared vision ensures in the short and medium run that competitive thinking is put aside in favor of cluster thinking and that clusters are implemented which build on a certain potential already existing in the economy. On the other hand, it ensures that in the long run, the continuation of a cluster is also guaranteed without financial support through subsidies from cluster policy~~ and~~. This also ensures that clusters can continue to develop independently and ~~adapted~~proactively adapt to the market without being exposed to the danger of possible misalignment or log-in through political requirements.

Place Leader - The Initiator of a Shared Vision

Place ~~leadership~~Leadership is defined as “the mobilization and coordination of diverse groups of actors to achieve a collective effort aimed at enhancing the development of a specific place” (Sotarauta 2021, p.152). In contrast to the association of a leader as “the one who makes it happen”, where leadership is viewed as an individual ability to direct others on what to do based on strong hierarchical relationships in decision making and formal power, ~~place leadership~~Place Leadership is often referred to as shared, cooperative, or collaborative due to the challenge of dealing with a variety of actors and stakeholders (Horlings et al., 2018). Place ~~leaders~~Leaders are usually not assigned leaders by position but leaders by personality. Place ~~leaders~~Leaders do not hold a formal position that allows them to execute power over others. They gain~~ their~~ power and leadership positions by influencing and convincing others (Sotarauta 2016; Beer et al. 2019). It is important to note that in addition to individuals from various sectors, including government, business, academia, and the community, institutions and organizations can also take on the role of Place Leader. Many institutions and organizations play such a leading role in shaping the development and growth of a particular place. For example, a chamber of commerce, local economic

development agency, or non-profit organization can take a leadership role by advocating for policies and investments that support the development of the place they serve, fostering collaboration and partnerships among stakeholders, and supporting a shared vision for the future of the place.. Place Leaders are widely believed to have an important impact on the economic and social performance of regions, for example, by helping unlocking a region from its path and guiding it in new directions (Sotarauta and Beer, 2017).

Place leadersLeaders gain their influence by stimulating imagination, (re)framing issues, and developing new agendas, helping to "think the unthinkable" (Horlings et al., 2010). Rather than trying to convince all stakeholders of the one "right and true" view, place leadersPlace Leaders strive to create a shared vision for the place among all stakeholders. They work as a kind of connector between the different stakeholders (Horlings et al., 2018) and try to strengthen the sense of place and identification by means ofutilizing a shared vision and thus develop the place better. Place leadersLeaders moderate the process of creating a shared vision among all cluster members. In this way, they indirectly influence the framework for implementing and managing resilient clusters.

Conclusion

The experience of the past years has shown that cluster policy, as it has been pursued in many cases in recent years, often does not lead to sustainably successful clusters. The "watering-can-like" approach of industry- and technology-specific cluster support programs without a specific focus on the cluster potential available in the regions often led to misallocations of clusters, with the result that these were discontinued when the state subsidies expired. To make cluster policy fittailored and thus sustainable, it would be necessary to identify a particular cluster or cluster potentials and its components within a local economy and develop a picture of the nature of the interactions among its actors, as well as an understanding of the nature of the market failures it faces (Burfitt and MacNeill, 2008). However, this

poses significant and almost insurmountable challenges for policymakers due to a lack of information and metrics.

Governments pursuing cluster policies should shift their attention from targeting and subsidizing specific industries and technologies to facilitating the development and functioning of clusters in the economy, thus also ~~taking into account~~considering the Commission of the European Communities' (17~~.10.~~ Oct 2008) demands on cluster policies. In the literature, this desired change in policy orientation is described as a shift from specific to general policy, ~~a shift~~ from direct to indirect intervention, and ~~a shift~~ from vertical to horizontal policy (see Chang 1994; McDonald and Dearden 2005). In essence, it should be left to the market itself to determine which clusters are formed regionally. After all, clustering is ultimately the result of entrepreneurial activity and is driven by the production of goods and services to generate profits. The state cannot sufficiently replace the market in the formation of these clusters (Sautet, 2002).

Resilient clusters are not created by cluster policies that intervene in the market. What is needed for a cluster to be sustainably successful, which also means adapting to the changing demands of the market, is a shared vision among the cluster members. Only through ~~the~~ sharing ~~of~~ a vision, the consequent recognition of the benefits of the cluster initiative ~~are the~~will be that companies ~~that are~~ often competing outside the cluster project ~~willing to set aside this thinking and thus~~are willing to open up to the cluster members. A ~~common~~shared vision is the only way to create a ~~sustainable,~~ trust-based network structure~~. —~~ and only on trust can we build up sustainable cluster activities. Therefore, the necessary knowledge spillovers within the cluster can occur and the cluster can ~~be thriving~~thrive. Especially in times when regional economies are faced with the task of transforming their economic structures due to recurring economic crises, globalization, digitalization, climate change, the depletion of natural resources and a variety of other societal dynamics such as migration and demographic change and, more recently, the ~~Corona~~COVID-19 pandemic or ~~the war between Russia and Ukraine~~belicistic conflicts among neighbor countries, clusters can make a significant contribution to ensuring that a region successfully overcomes these challenges. A shared vision, the associated open-mindedness toward new technologies

and the opportunity for intensive exchange and resulting innovations not only lead the way to the resilience of clusters, but also to the sustainable economic success of regions.

The process of establishing and implementing a common vision is facilitated by ~~place leaders~~Place Leaders, who are the link between the various interests of the cluster members. Since ~~place leaders~~Place Leaders have no formal power and can ~~therefore~~ neither pass guidelines nor create monetary incentives, ~~it is almost impossible that~~ clusters ~~are initiated~~operated by Place Leaders will have the critical qualitative mass to initiate and ~~promoted without lasting substance~~promote sustainable networks as they are not merely attracted by false or misleading incentives.

In conclusion, future cluster policy should return to the horizontal measures of cluster promotion and not take an active role in the initiation of clusters. Future scientific work should consider the importance of a shared vision among cluster members when analyzing cluster formation, development and governance. It should also be questioned to what extent effective ~~place leadership~~Place Leadership and a shared vision could outperform current policy measures in other regional, industrial and structural areas and might even make them redundant.

Conflict of interest

On behalf of all authors, the corresponding author states that there is no conflict of interest.

References

Formatiert: Deutsch (Deutschland)

Ahn, Y.-J. (2018): Clusterförderung und -politik in Deutschland. In: *Journal of the Economic Geographical Society of Korea* 21, 425–437. DOI: 10.23841/egsk.2018.21.4.425.

Beer, A., Ayres, S., Clower, T., Faller, F., Sancino, A~~.~~., and Sotarauta, M. (2019): Place leadership and regional economic development: a framework for cross-regional analysis. In: *Regional Studies* 53~~ ~~(2), 171–182. DOI: 10.1080/00343404.2018.1447662.

Formatiert: Englisch (Vereinigtes Königreich)

Blümel, C. (2021): Innovations in innovation policy: reconstructing the emergence, legitimation and dynamics of cluster policies in Germany. In*: Innovation: The European Journal of Social Science Research* 34(4), 533-559. DOI: 10.1080/13511610.2020.1798216

Bowden, A~~.~~., and Liddle, J. (2018): Evolving public sector roles in the leadership of place-based partnerships: from controlling to influencing policy? In: *Regional Studies* 52~~ ~~(1), 145–155. DOI: 10.1080/00343404.2017.1346369.

Boyatzis, R. E., Rochford, K., and Taylor, S. N. (2015): The role of the positive emotional attractor in vision and shared vision: toward effective leadership, relationships, and engagement. In: *Frontiers in Psychology* 6~~:~~, 670. DOI: 10.3389/fpsyg.2015.00670.

Bührer, S., Heinze, T., and Kuhlmann, S. (2003): Lassen sich Innovationsnetzwerke politisch erschaffen? Aufgaben der Evaluation von Multi-Akteur-/Multi-Maßnahmen-Initiativen. In: *Politische Steuerung von Innovationssystmen? Potenziale der Evaluation von Multi-Akteur-/Multi-Maßnahmenprogrammen*, ed. S. Bührer and S. Kuhlman, Stuttgart: Fraunhofer IRB Verlag, 3-21

Burfitt, A., and MacNeill, S. (2008): The challenges of pursuing cluster policy in the congested state. In: *International Journal of Urban and Regional Research* 32~~ ~~(2), 492–505. DOI: 10.1111/j.1468-2427.2008.00784.x.

Chang, H.-J. (1994): The political economy of industrial policy. New York: St. Martin's Press, Inc.

Chen, C.-L.; Lin, Y.-C.; Chen, W.-H., and Heng, X.-S. (2018): Determinants of cluster leadership and identification on cluster innovation model. In: *Leadership & Organization Development Journal* 39~~ ~~(4), 538–553. DOI: 10.1108/LODJ-10-2017-0305.

Commission of the European Communities (17~~.10.~~ Oct 2008): *Communication from the Commission to the Council, the European Parliament, the European Economic and Social Committee and the Committee of the Regions. Towards world-class clusters in the European Union: Implementing the broad-based innovation strategy*. {SEC(2008) 2637}. Brussels.

Cumbers, A~~.~~., and MacKinnon, D. (2004): Introduction: Clusters in Urban and Regional Development. In: *Urban Studies* 41~~ ~~(5/6), 959–969.

Graf, H., and Broekel, T. (2020): A shot in the dark? Policy influence on cluster networks. In: *Research Policy* 49(3), 103920. DOI: 10.1016/j.respol.2019.103920.

Grashof, N. (2020): Putting the watering can away. Towards a targeted (problem-oriented) cluster policy framework. *Papers in Innovation Studies*, 2020/4, Lund University, CIRCLE - Centre for Innovation Research.

Formatiert: Deutsch (Deutschland)

Griffin, M. A., Parker, S. K., and Mason, C. M. (2010): Leader vision and the development of adaptive and proactive performance: A longitudinal study. In: *Journal of Applied Psychology* 95(1), 174–182. DOI: 10.1037/a0017263.

Horlings, L., Ostaaijen, J.J.C., and Stoep, H. (2010): Vital coalitions, vital regions: cooperation in sustainable, regional development. Wageningen Academic Publishers.

Horlings, L., Roep, D., and Wellbrock, W. (2018): The role of leadership in place-based development and building institutional arrangements. In: *Local Economy* 33(3), 245–268. DOI: 10.1177/0269094218763050.

Hospers, G.-J., Desrochers, P., and Sautet, F. (2008): The Next Silicon Valley? On the Relationship between Geographical Clustering and Public Policy. In: *International Entrepreneurship and Management Journal* 5, 285–299. DOI: 10.1007/s11365-008-0080-5.

Jankowiak, A. H. (2019): The cluster policy status in economic policies of selected countries. Comparative approach, *The 13th International Days of Statistics and Economics: Conference Proceedings.* Prague, 5-7 September. 576-584, DOI:10.18267/pr.2019.los.186.57.

Kowalski, A, M. (2020). Towards an Asian Model of Clusters and Cluster Policy: The Super Cluster Strategy. In: *Journal of Competitiveness* 12(4), 74–90. DOI: 10.7441/joc.2020.04.05.

Kozonogova, E., Elokhova, I., Dubrovskaya, J., and Goncharova, N. (2019): Does state cluster policy really promote regional development? The case of Russia. In*: IOP Conference Series: Materials Science and Engineering* 497(1), 12044. DOI: 10.1088/1757-899X/497/1/012044.

Lehmann, E. E., and Menter, M. (2018): Public cluster policy and performance. In: *The Journal of Technology Transfer* 43(3), 558-592. DOI: 10.1007/s10961-017-9626-4.

Martin, R., and Sunley, P. (2003): Deconstructing clusters: chaotic concept or policy panacea? In: *Journal of Economic Geography* 3(1), 5–35. DOI: 10.1093/jeg/3.1.5.

McDonald, F., and Dearden, S. (2005): *European economic integration*. 4th Ed. Financial Times Prentice Hall.

Moriano, J. A., Molero, F., Topa, G., and Lévy Mangin, J.-P-. (2014): The influence of transformational leadership and organizational identification on intrapreneurship. In: *International Entrepreneurship and Management Journal* 10(1), 103–119. DOI: 10.1007/s11365-011-0196-x.

Norton, R. (1999). Cluster theories: Spatial externalities. In: *The web book of regional science*. ed. R. W. Jackson. West Virginia University: Regional Research Institute.

Porter, M. E. (1998): On Competition. A Harvard Business Review Book. Boston, Harvard Business School Publishing.

Porter, M.E., and Stern, S. (2001): Innovation: Location Matters. In: *MIT Sloan Management Review* 42 (4), 28–36.

Rothgang, M., and Lageman, B. (2011): Innovationspolitischer Mehrwert durch Vernetzung?: Cluster- und Netzwerkförderung als Politikinstrument auf Bundes- und Länderebene. In: *Quarterly Journal of Economic Research* 80, 143–166. DOI: 10.3790/vjh.80.3.143.

Sautet, F. (2002): Kirznerian Economics: Some Policy Implications and Issues. In: *Journal des Economistes et des Etudes Humaines* 12:, 131-151. DOI: 10.2202/1145-6396.1053.

Schmitz, H., and Nadvi, K. (1999): Clustering and Industrialization: Introduction. In: *World Development* 27 (9), 1503–1514.

Sotarauta, M. (2016): Place leadership, governance and power. In: *Administration* 64 (3-4), 45–58. DOI: 10.1515/admin-2016-0024.

Sotarauta, M. (2021): Combinatorial Power and Place Leadership. In: *Handbook on City and Regional Leadership*, 152–166. Cheltenham.

Sotarauta, M.., and Beer, A. (2017): Governance, agency and place leadership: lessons from a cross-national analysis. In: *Regional Studies* 51 (2), 210–223. DOI: 10.1080/00343404.2015.1119265.

Sotarauta, M., Beer, A.., and Gibney, J. (2017): Making sense of leadership in urban and regional development. In: *Regional Studies* 51 (2), 187–193. DOI: 10.1080/00343404.2016.1267340.

Steiner, M. (1998): The discreet charm of clusters: an introduction. In: M. Steiner (Ed.): *Clusters and regional specialisatio*n, 1-17. London: Pion.

Wolf, C. (1990): Markets or governments: Choosing between imperfect alternatives. *Cambridge, MA:* The MIT Press

Zenker, A., Delponte, L., Mayán, N. D., Wintjes, R., Amichetti, C., Carneiro, J., et al. (2019): *Cluster programmes in Europe and beyond. European Observatory for Clusters and Industrial Change*. Available at: https://clustercollaboration.eu/sites/default/files/news_attachment/cluster_programmes_in_europe_and_beyond_0.pdf, last retrieved: 09.04.2023.

Formatiert: Englisch (Vereinigtes Königreich)
